# Epidemiological study of renal involvement in sarcoidosis in Japan using the Japan Renal Biopsy Registry

**DOI:** 10.1007/s10157-025-02688-7

**Published:** 2025-04-30

**Authors:** Yoshinori Kamata, Tsuneo Konta, Toshiaki Nakano, Naoko Masuzawa, Kazunobu Ichikawa, Takaya Ozeki, Shoichi Maruyama, the Committee for Renal Biopsy, Disease Registry of the Japanese Society of Nephrology

**Affiliations:** 1 Department of Public Health and Hygiene, Yamagata University Graduate School of Medicine, 2-2-2 Iida-Nishi, Yamagata, 990-9585 Japan; 2 Department of Medicine and Clinical Science, Graduate School of Medical Sciences, Kyushu University Center for Cohort Studies, Fukuoka, Japan; 3 Department of Pathology, Otsu City Hospital, Shiga, Japan; 4 Department of Cardiology, Pulmonology, and Nephrology, Faculty of Medicine, Yamagata University, Yamagata, Japan; 5 Department of Nephrology, Nagoya University Graduate School of Medicine, Nagoya, Japan

**Keywords:** Sarcoidosis, Renal sarcoidosis, Renal biopsy, Tubulointerstitial nephritis, Epidemiology

## Abstract

**Background:**

In sarcoidosis, renal involvement is less common than lung involvement. Epidemiology and clinical characteristics of the patients with renal involvement are largely unknown.

**Methods:**

From the database of the Japan Renal Biopsy Registry (J-RBR), the patients who were diagnosed with renal involvement of sarcoidosis such as sarcoidosis-related tubulointerstitial nephritis or renal calcinosis were extracted. The prevalence and clinical characteristics of these cases were analyzed. After removing the cases with concomitant glomerular diseases or renal calcinosis, the correlation between urinary protein-creatinine ratio (UPCR) and eGFR or serum albumin levels were evaluated by Pearson’s correlation (r). In addition, distributions of serum albumin levels were compared across the categories of urinary albumin levels.

**Results:**

Among the 55,885 participants in the J-RBR database, 135 patients (66 men and 69 women, 0.24% of total registration) had renal involvement of sarcoidosis including nine cases with concomitant glomerular diseases: nephrosclerosis (*n* = 4), IgA nephropathy (*n* = 2), diabetic nephropathy (*n* = 2) and unclassified (*n* = 1). Mean value (± standard deviation) of age was 62 ± 14, eGFR was 30.7 ± 15.2, and UPCR was 0.50 ± 0.68. Among the patients without any concomitant glomerular disease or renal calcinosis, UPCR was negatively correlated with eGFR (r = ** − **0.531, *p* < 0.001) and serum albumin level (r = ** − **0.352, *p* < 0.001).

**Conclusion:**

Data from a nationwide biopsy registry revealed that main forms of histopathology of renal involvement of sarcoidosis was tubulointerstitial nephritis and these cases were common in older adults. The intensity of the tubulointerstitial nephritis may be related to the degree of renal dysfunction and proteinuria.

## Introduction

Sarcoidosis is a systemic inflammatory disease with unknown etiology, characterized by non-caseating granulomas with a predilection for the lungs, lymph nodes, skin, and eyes. This tends to occur in women and before the age of 50 years [1]. In 1999, the Steering Committee of the Case–Control Etiologic Study of Sarcoidosis (ACCESS) developed a sarcoidosis assessment instrument and described its renal involvement as nephrolithiasis, granulomatous kidney disease, or steroid-responsive renal failure [2]. Renal involvement is rare in sarcoidosis. In a study of 736 cases of biopsy-proven sarcoidosis in the U.S. between 1997 and 1999, only five had renal involvement (0.7% of all cases) [3]. In Japan, a study by Sawahata et al. of 588 cases of sarcoidosis between 1974 and 2012 [4] (431 histologically proven and 157 clinically diagnosed cases) found renal involvement in six cases (1.0% of all cases). From the standpoint of renal biopsy, previous studies reported that the prevalence of sarcoidosis-related renal lesions was 0.1–0.6% in Western countries [5–7] and Eastern Japan [8], and the number of patients with biopsy-proven sarcoidosis-related tubulointerstitial lesions analyzed in the largest case series is 27 [6]. The epidemiology and clinical characteristics of patients with renal involvement have not been well described because of the limited number of applicable cases. The largest case series described the epidemiology of 47 patients (30 men) with sarcoidosis-related tubulointerstitial nephritis; however, the population that underwent renal biopsy was unknown [9]. The Japan Renal Biopsy Registry (J-RBR) is a nationwide renal biopsy database established in 2007 by the Japanese Society of Nephrology [10], and it collects the cross-sectional data of the patients at the time of biopsy. As of December 2021, the J-RBR had 55,885 registered participants, making it the world’s largest database system for renal biopsies. Therefore, we conducted this study to clarify the clinical characteristics of the patients with renal involvement in sarcoidosis using the data from the largest renal biopsy database.

## Materials and methods

### Registration system of the J-RBR

Data from the J-RBR were used in this study. The J-RBR has enrolled patients who underwent renal biopsy in Japan since 2007. The original 2007 J-RBR system registered the diagnosis of participants based on three components, as proposed by the World Health Organization (WHO) [11, 12]: (i) clinical diagnosis, (ii) histological diagnosis by pathogenesis, and (iii) histological diagnosis by histopathology. In 2018, the registration system was updated [13]. The revised 2018 J-RBR system was designed to register participants based on an etiology-focused diagnostic classification that encompassed almost all known kidney diseases in 22 categories: 18 glomeruli, one tubulointerstitial nephritis, one hereditary congenital, one transplant-related, and one other.

### Selection of eligible participants

According to the American Thoracic Society statement on sarcoidosis in February 1999, the typical histology of renal involvement is tubulointerstitial nephritis or nephrocalcinosis [14]. The patients with histology applicable to the J-RBR were extracted for this study based on this statement. The original 2007 J-RBR system did not have any specific registration categories for sarcoidosis. Therefore, sarcoid-related cases registered between 2007 and 2017 were selected through a text search based on free descriptions of diagnosis. From the 2007 system database, 104 of the 38,351 participants were registered with any descriptions of sarcoidosis. Among the 104 cases, 28 patients without tubulointerstitial lesions and one repeat biopsy case were excluded, then 74 patients with tubulointerstitial nephritis and one with renal calcinosis were included to the analysis. Regarding the excluded 28 cases, five cases had glomerulosclerosis, five cases had minor glomerular changes, four cases had membranous nephropathy and the other 14 cases were unknown.

**Fig. 1:**
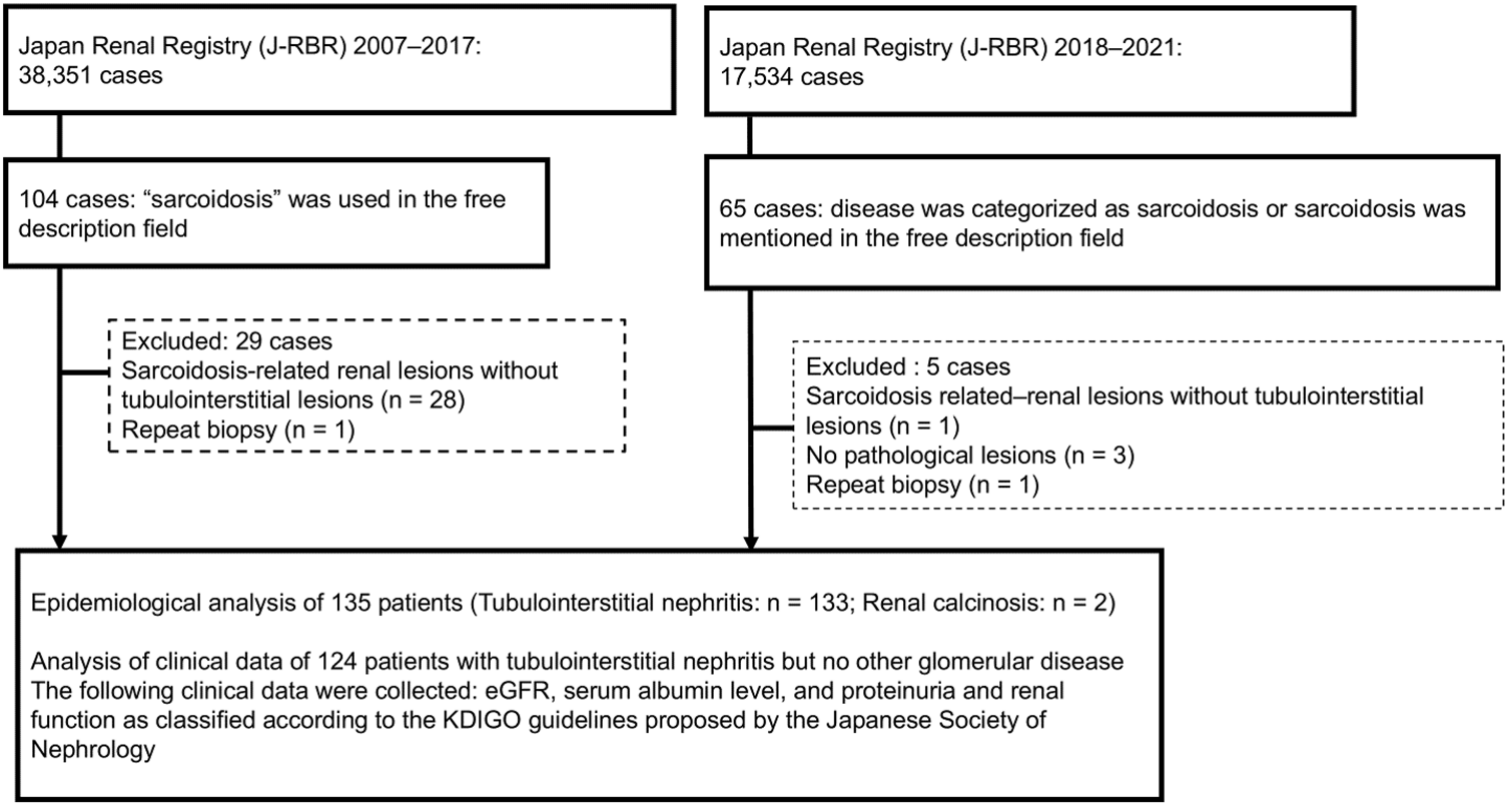
Flowchart of selection of sarcoidosis-related cases from the J-RBR

In the 2018 system, renal involvement in sarcoidosis can be specified from the category titled “tubulointerstitial nephritis/sarcoidosis” in the list of disease categories. Additionally, text search for “sarcoidosis” was also performed in the free descriptions for each case. Of the 17,534 participants registered in the J-RBR between 2018 and 2021, 65 were registered as renal involvement in sarcoidosis. The patients with diabetic nephropathy without tubulointerstitial lesions (n = 1), repeat biopsies (*n* = 1), or no remarkable findings (*n* = 3) were excluded. Thus, 60 patients with tubulointerstitial nephritis (*n* = 59) or renal calcinosis (*n* = 1) were included. Combining the patients from the 2007 and 2018 systems, 135 patients registered in the J-RBR between 2007 and 2021 were included (Fig. 1). Histological diagnosis including tubulointerstitial nephritis or renal calcinosis depended on the selection for the standardized diagnosis classifications or free description by the registered institutions.

### Clinical data

The data analyzed in this study included sex, age, estimated glomerular filtration rate (eGFR) at biopsy, serum albumin level, urinary protein level, presence of hematuria, and the presence of diabetes mellitus. The eGFR was calculated using a formula optimized for the Japanese population [15]. The J-RBR database does not collect any information of patient’s race or ethnicity. Proteinuria and renal function were classified according to the Kidney Disease Improving Global Outcomes guidelines [16] arranged by the Japanese Society of Nephrology. Renal function was classified as GFR category G1 (G1) for values ≥ 90 mL/min/1.73m^2^, G2 for 60–89 mL/min/1.73m^2^, G3a for 45–59 mL/min/1.73m^2^, G3b for 30–44 mL/min/1.73m^2^, G4 for 15–29 mL/min/1.73m^2^, and G5 for < 15 mL/min/1.73m^2^. In Japan, urinary albumin levels are not commonly measured but rather urinary protein levels. Therefore, the UPCRs of the patients were classified as albuminuria category A1 for values < 0.15 g/gCr, A2 for 0.15–0.49 g/gCr, and A3 for ≥ 0.5 g/gCr. Microscopic hematuria was defined as ≥ 5–9 red blood cells per strongly magnified field of view.

We described the epidemiological characteristics of the 135 eligible cases and analyzed the clinical data, focusing on the 124 cases with tubulointerstitial nephritis, excluding nine cases complicated by glomerular diseases and cases of two renal calcinosis, to determine the pure effect of tubulointerstitial nephritis on proteinuria.

Data analyses were performed using IBM SPSS version 29. The Mann–Whitney U test was used to compare the means of the data between the two groups. Fisher’s exact test was used to determine the ratio of items between the two groups. The correlation analysis between the UPCR, eGFR, and serum albumin level was performed using Pearson’s correlation coefficient (r). The Tukey–Kramer test was used to compare the mean serum albumin levels among groups with modified albuminuria categories.

## Results

### Epidemiology and clinical characteristics

**Fig. 2:**
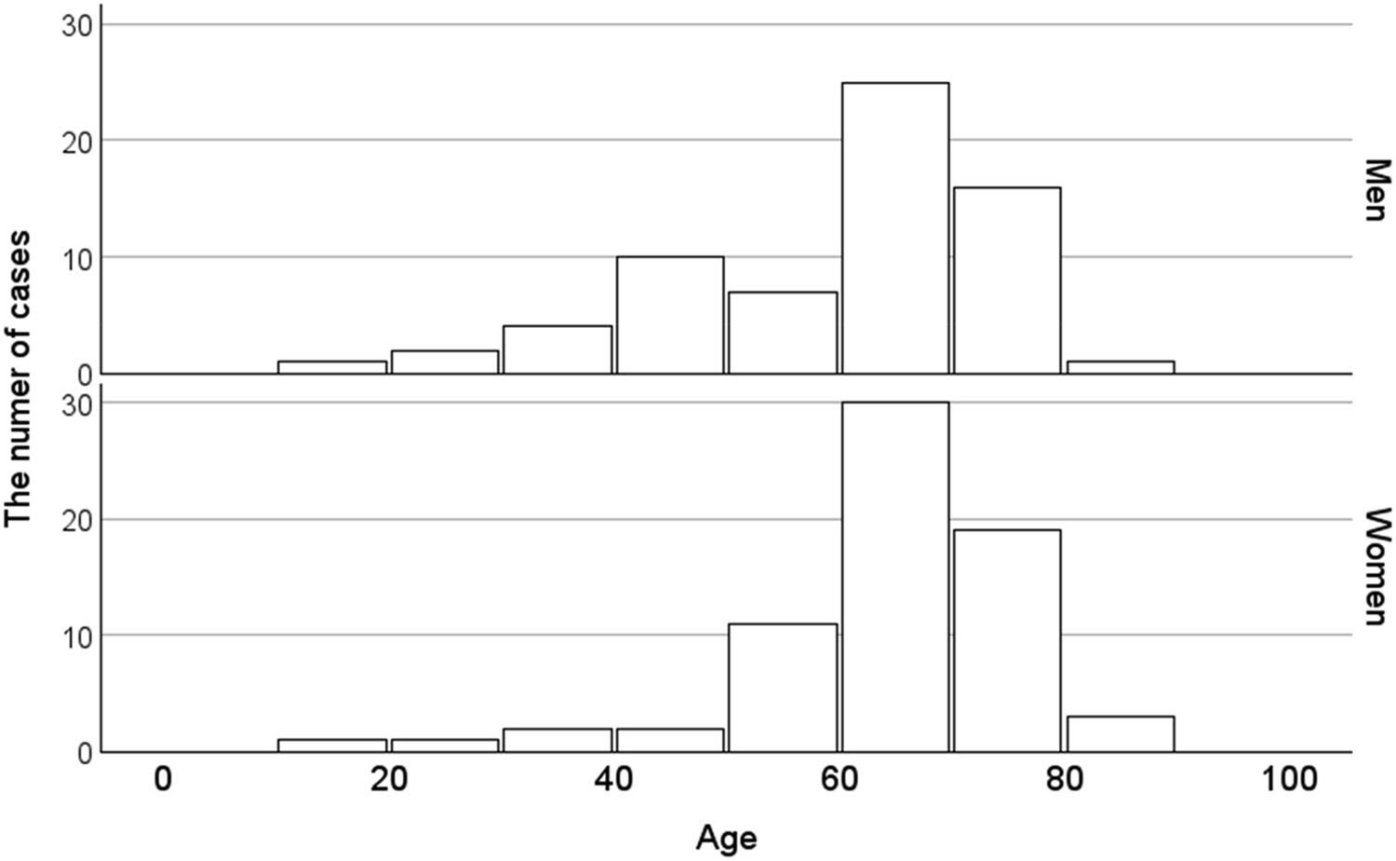
Histogram of sarcoid-related cases by age

We included 135 cases with renal involvement in sarcoidosis, which accounted for 0.24% of the total number of J-RBR participants (2007–2017: 75 cases, 0.20%; 2018–2021: 60 cases, 0.34%). Regarding sarcoidosis-associated tubulointerstitial nephritis, in the original 2007 J-RBR system, tubulointerstitial nephritis was classified into acute tubular necrosis/tubulointerstitial nephritis (ATN/TIN) and the total number was 1031. The cases with sarcoidosis associated tubulointerstitial nephritis accounted for 7.2% of all cases with ATN/TIN. In the revised 2018 J-RBR system, tubulointerstitial nephritis were classified into tubulointerstitial nephropathy/tubulointerstitial nephritis or nephropathy associated with connective tissue diseases/tubulointerstitial nephritis and the total number of tubulointerstitial nephritis was 686. The cases with sarcoidosis associated tubulointerstitial nephritis accounted for 8.6% of all cases with tubulointerstitial nephritis. The number of biopsy-proven renal involvement in sarcoidosis in the J-RBR was slightly higher in women (69 cases, 51%). The mean age was 59 ± 15 years (median 64, interquartile range 48–70) for men and 64 ± 12 years (median, 64 interquartile range 60–71) for women, and the mean eGFR was 32.2 ± 14.1 mL/min/1.73 m^2^ for men and 29.2 ± 16.3 mL/min/1.73 m^2^ for women. Eighteen patients (13%) had diabetes mellitus (Table 1). Severe renal dysfunction (GFR category G4 or G5) affected the majority of patients (71/133, 53.4%) and was more common in women (Table 2). In terms of the number of cases by age, the highest number of patients was in their 60 s (*n* = 55). The highest number of cases was found in both male and female patients in their 60 s (25 men and 30 women), and the small peak of number of cases was found among men in their 40 s (Fig. 2). Tubulointerstitial nephritis was the main histological feature of sarcoidosis-related renal involvement in of 133/135 (98.5%) cases. Nine patients had concomitant glomerular lesions, including four with nephrosclerosis due to hypertension/arteriosclerosis, two with IgA nephropathy, two with diabetic nephropathy, and one with glomerular disease other than IgA nephropathy (Table 3). One patient with tubulointerstitial nephritis had Sjögren’s syndrome. The mean UPCR was 0.50 ± 0.68 g/gCr. The albuminuria category was evaluated in 130 patients. Of these, 33 (25.4%) were classified as A1, 64 (49.2%) as A2, and 33 (25.4%) as A3 (Table 4). Microhematuria was observed in 15 (11%) patients (Table 1). In the albuminuria category, microhematuria was most frequently observed in patients classified as having albuminuria A3. Regarding histological lesions, microhematuria was observed in 14 cases of tubulointerstitial nephritis and one case of tubulointerstitial nephritis and concomitant IgA nephropathy.

**Table 1:**
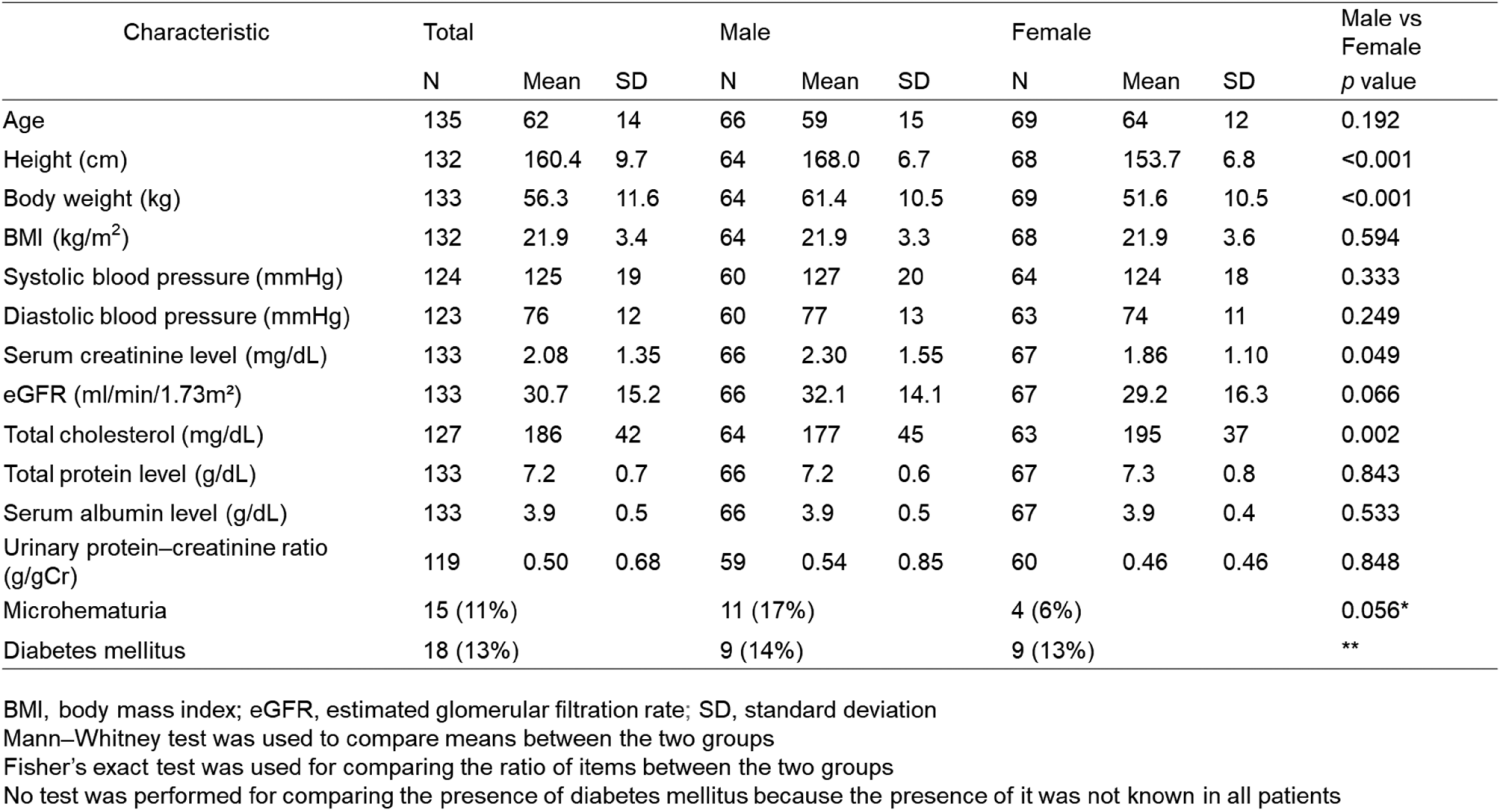
Characteristics of the patients with sarcoid-related renal disease

**Table 2:**
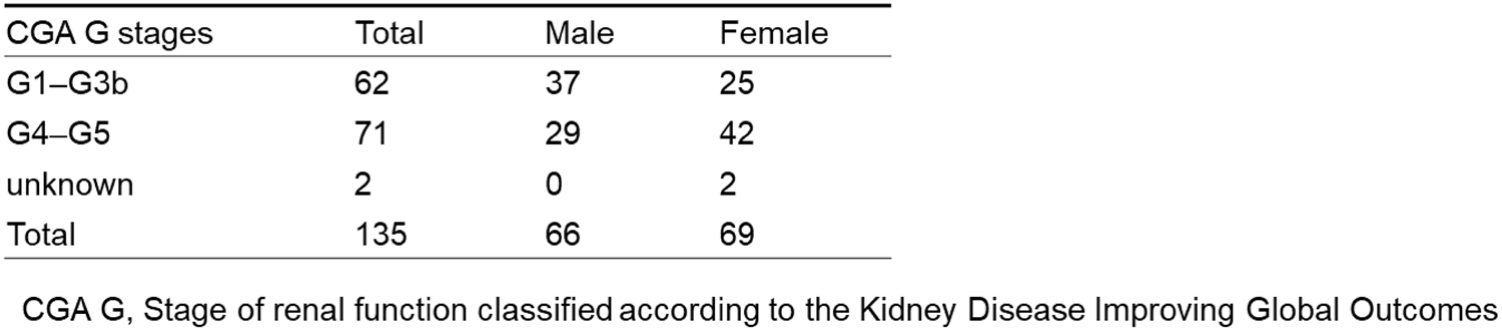
Chronic kidney disease stage by patient sex

**Table 3:**
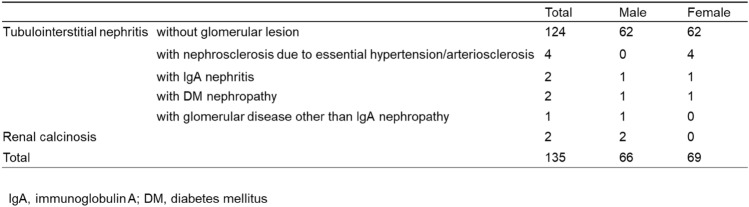
Histological classification in the patients with sarcoid-related renal disease

**Table 4:**
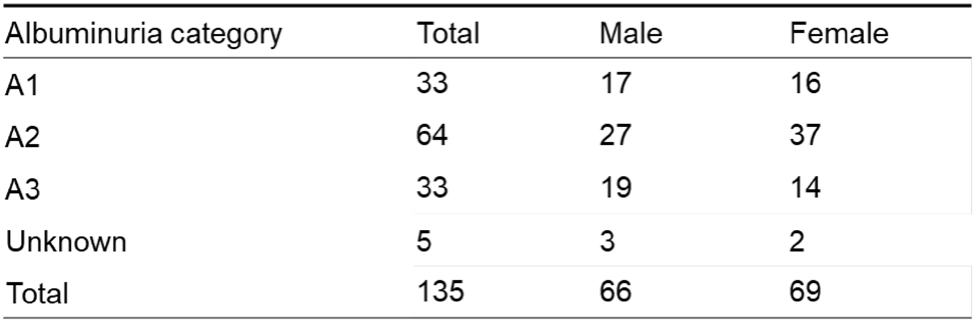
Number of patients in each albuminuria category by sex

### Association between eGFR, UPCR, and serum albumin level

The relationship between laboratory data, including UPCR, eGFR, and serum albumin level, was analyzed in 124 patients without any concomitant glomerular diseases.

**Fig. 3:**
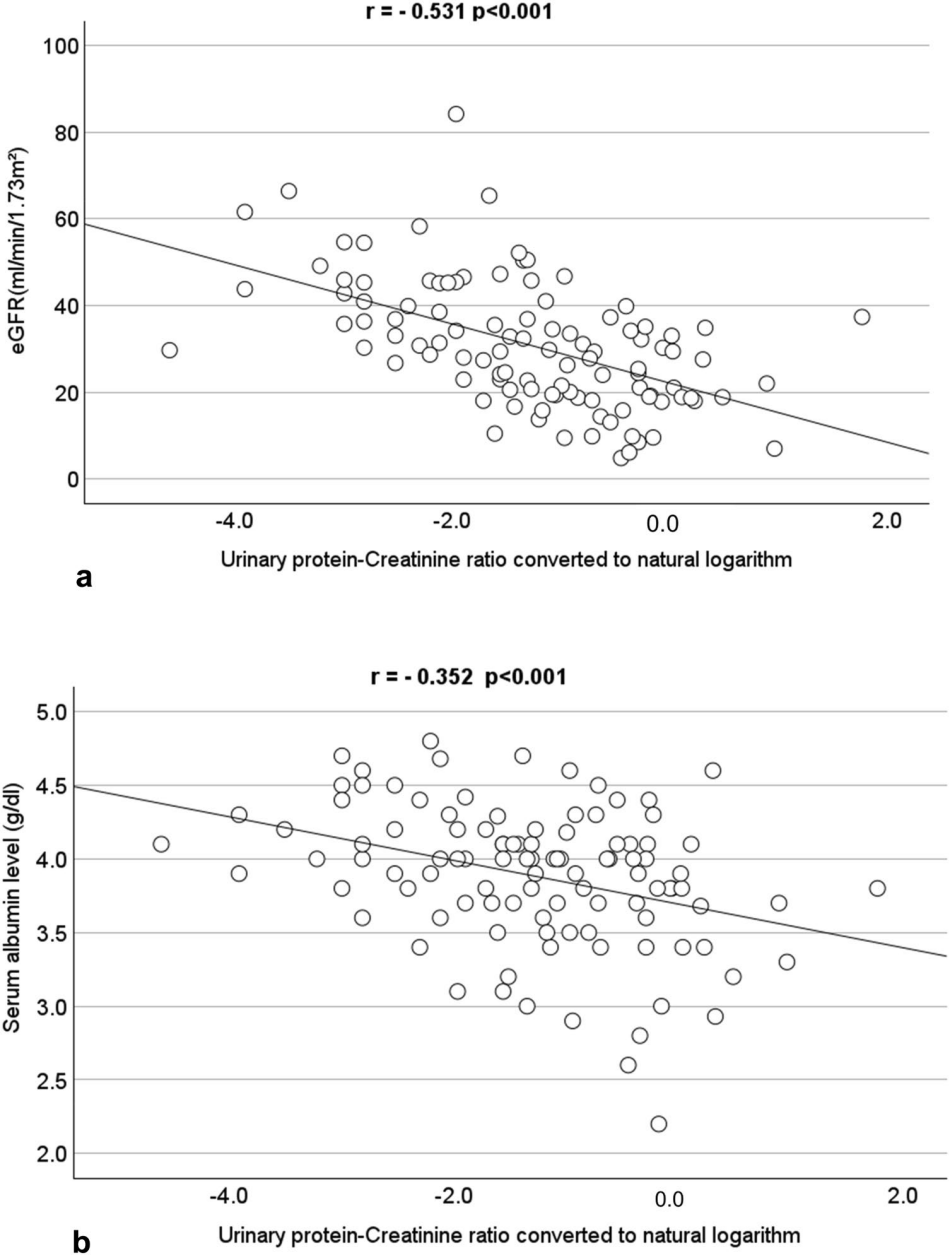
Urinary protein-creatinine ratio converted to natural logarithm negatively correlated with **a** eGFR and **b** serum albumin level in Pearson’s correlation

**Fig. 4:**
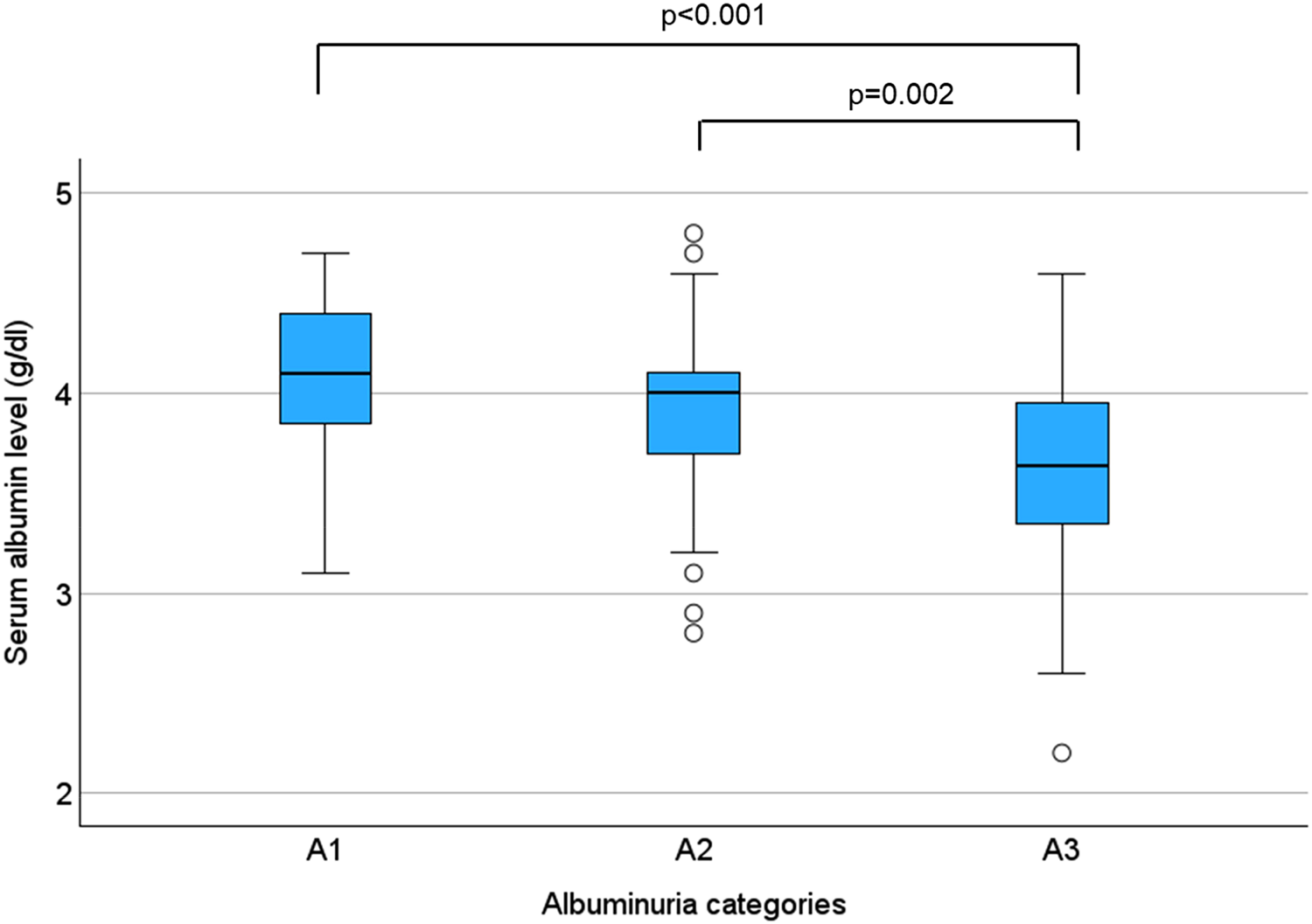
Serum albumin levels by albuminuria category. Tukey–Kramer test revealed significant differences between A1 and A3 and between A2 and A3

UPCR converted to a natural logarithm was negatively correlated with eGFR (r = ** − **0.531, *p* < 0.001) (Fig. 3a) and negatively correlated with serum albumin levels (r = ** − **0.352, *p* < 0.001) (Fig. 3b). We compared the mean serum albumin levels by the arranged albuminuria category: the serum albumin level was 4.06 ± 0.42 g/dL, 3.94 ± 0.40 g/dL, and 3.58 ± 0.54 g/dL for category A1, A2, and A3, respectively. The Tukey–Kramer test was performed on serum albumin levels in the three groups and a statistically significant difference in serum albumin levels was found between A1 and A3 and between A2 and A3, with *p* < 0.001 and *p* = 0.002, respectively (Fig. 4).

## Discussion

In this study, we used the data from the J-RBR, the largest Japanese kidney biopsy database system, to clarify the epidemiological and clinical characteristics of patients with sarcoidosis-related renal involvement. In the largest case series reported in France, 62% of patients had advanced renal failure, 66% had proteinuria, and 22% had hematuria [9]above. In our cases, compared to the data in France, there was a lower percentage of advanced renal failure, a higher percentage of proteinuria and a lower percentage of microhematuria. The age histogram of sarcoidosis-related renal involvement demonstrated using the J-RBR showed a main peak in renal biopsy cases in the 60 s and 70 s age groups for both male and female patients, and a small peak among male patients in their 40 s. The main peak of the age histogram in sarcoidosis-related renal involvement in the J-RBR was higher than that of patients with sarcoidosis at diagnosis in previous epidemiological studies. The ACCESS epidemiological study in the US reported that the peak age at diagnosis of sarcoidosis was in the 35–39 s for both sexes [3]. According to Japanese epidemiological studies, the predominant age group at the time of diagnosis of sarcoidosis is bimodal, with the first peak in the 50–60 s age group and the second peak in the 20–30 s [4, 17]. In previous studies [7, 18–20], biopsy-proven renal involvement of sarcoidosis was diagnosed at the same time or later than the new diagnosis of sarcoidosis, which might contribute to higher age distribution of patients with biopsy-proven sarcoidosis-related renal involvement than that of patients newly diagnosed with sarcoidosis. According to the previous epidemiological studies on sarcoidosis, renal involvement in sarcoidosis is rare; however, autopsy reports of sarcoidosis show renal involvement in 19% of cases [21]. These findings suggest that sarcoidosis-related renal involvement have been underestimated.

After the revision of registration system of the J-RBR, the percentage of sarcoidosis-related cases with tubulointerstitial lesions increased from 0.20% in 2007 system to 0.34% in 2018 system. The addition of sarcoidosis as a new sub-item of tubulointerstitial nephropathy in the diagnostic classification might be a factor in the increased registration of sarcoidosis-related renal involvement.

We found that some glomerular diseases or glomerular lesions, such as nephrosclerosis, IgA nephropathy, diabetic nephropathy, co-existed with tubulointerstitial nephritis in the J-RBR. There are several references which can support that certain glomerular diseases can be overlapped with renal involvement in sarcoidosis. Recently, an association between sarcoidosis and atherosclerosis has been reported. Yong et al. suggested that sarcoidosis should be associated with arterial stiffness evaluated by pulse wave velocity in their review [22]. Yilmaz et al. showed the presence of endothelial dysfunction and subclinical atherosclerosis evaluated by carotid intima-media thickness and flow-mediated dilatation measured by ultrasoundsonography [23]. Selendili et al. proposed that atherogenic index calculated by lipid profiles and intimal thickness might be a predictor for atherosclerosis in patients with sarcoidosis [24].]. Löffler et al. reported IgA nephropathy in nine of 27 cases of renal biopsy related to sarcoidosis [25]. Some case reports of IgA nephropathy associated with sarcoidosis have been published [26, 27]. Stehle et al. found that IgA nephropathy, membranous nephropathy, minimal change nephrotic syndrome, and focal segmental glomerulosclerosis were concomitant with granulomatous tubulointerstitial nephritis [18]. However, the mechanism underlying the association between sarcoidosis and concomitant glomerular diseases remains unclear. Benmelouka et al. reported a higher prevalence of diabetes mellitus in patients with sarcoidosis than in healthy controls based on a meta-analysis in North America, Europe, and Asia [28]; however, they could not identify the timing of diabetes mellitus diagnosis or follow-up period with corticosteroid therapy based on their review and meta-analysis, and the relationship between diabetes mellitus and sarcoidosis remains unclear.

Correlation analysis showed that a higher UPCR was negatively associated with a lower eGFR and serum albumin level. Mean serum albumin level in the higher-grade albuminuria category was lower. These results can provide suggestions as following: 1) a greater degree of inflammation by tubulointerstitial nephritis leads to both higher urinary protein excretion and lower renal function; and 2) a decrease in albumin reabsorption, as well as small molecular proteins, such as α₁-microglobulin and β₂-microglobulin, in the renal tubules due to tubulointerstitial nephritis results in a lower serum albumin level. The proximal tubules reabsorb albumin from the glomerular filtrates. Albumin reabsorption is mediated by endocytosis involving the megalin-cubilin complexes on S1 segment [29, 30]. However, the mechanism through which inflammatory tubulointerstitial injury affects albumin reabsorption in the tubulointerstitium remains unclear. As limitations of this study, we analyzed cross-sectional data collected at time of biopsy only from the J-RBR database. The database didn't include any markers of tubulointerstitial injury (urinary tubular protein, pathological estimation of tubulointerstitial fibrosis or tubular atrophy), chronological clinical data and information on medical treatment, clinical course and medical history. Pathological diagnosis is based on a system that selects from a comprehensive, standardized diagnostic panels and sub-items and it depends on registration details by each medical institution and we didn't directly access relevant renal biopsy specimen. We did not get the information about the initial diagnosis of sarcoidosis and extra-renal involvements of sarcoidosis. Therefore, adding these data, a longitudinal study is needed to determine how the initial data at the time of renal biopsy affect prognosis. 

## Conclusion

Here, we have done an epidemiological study on the patients with renal involvement in sarcoidosis using the J-RBR database with the largest sample size on renal biopsy patients ever. The median value of age was higher than that of overall sarcoidosis previously reported, which suggested that renal involvement of sarcoidosis be often diagnosed later than the initial diagnosis of sarcoidosis. Major histological findings were tubulointerstitial nephritis. From the analysis of the patients with tubulointerstitial nephritis, the levels of UPCR were negatively correlated with both eGFR and serum albumin level, which suggested that development of tubulointerstitial nephritis might relate to decreased albumin reabsorption by the damaged proximal tubules and subsequent hypoalbuminemia.

## Data Availability

The data that support the findings of this study are not openly available due to reasons of sensitivity and are available from the corresponding author upon reasonable request.
