# Correction: Epidemiological study of renal involvement in sarcoidosis in Japan using the Japan Renal Biopsy Registry

**DOI:** 10.1007/s10157-025-02702-y

**Published:** 2025-05-18

**Authors:** Yoshinori Kamata, Tsuneo Konta, Toshiaki Nakano, Naoko Masuzawa, Kazunobu Ichikawa, Takaya Ozeki, Shoichi Maruyama

**Affiliations:** 1 Department of Public Health and Hygiene, Yamagata University Graduate School of Medicine, 2-2-2 Iida-Nishi, Yamagata, 990-9585 Japan; 2 Department of Medicine and Clinical Science, Graduate School of Medical Sciences, Kyushu University Center for Cohort Studies, Fukuoka, Japan; 3 Department of Pathology, Otsu City Hospital, Shiga, Japan; 4 Department of Cardiology, Pulmonology, and Nephrology, Faculty of Medicine, Yamagata University, Yamagata, Japan; 5 Department of Nephrology, Nagoya University Graduate School of Medicine, Nagoya, Japan

**Correction: Clinical and Experimental Nephrology** 10.1007/s10157-025-02688-7

The article “Epidemiological study of renal involvement in sarcoidosis in Japan using the Japan Renal Biopsy Registry”, written by Yoshinori Kamata, Tsuneo Konta, Toshiaki Nakano, Naoko Masuzawa, Kazunobu Ichikawa, Takaya Ozeki, Shoichi Maruyama was originally published under © The Author(s), under exclusive licence to Japanese Society of Nephrology 2025. As a result of the subsequent decision to publish the article under the open access model, the article’s copyright notice was changed on 03 May 2025 to © The Author(s) 2025 and the article is now distributed under a Creative Commons Attribution 4.0 International License, which permits use, sharing, adaptation, distribution and reproduction in any medium or format, as long as you give appropriate credit to the original author(s) and the source, provide a link to the Creative Commons licence, and indicate if changes were made. The images or other third party material in this article are included in the article’s Creative Commons licence, unless indicated otherwise in a credit line to the material. If material is not included in the article’s Creative Commons licence and your intended use is not permitted by statutory regulation or exceeds the permitted use, you will need to obtain permission directly from the copyright holder. To view a copy of this licence, visit http://creativecommons.org/licenses/by/4.0.

